# Radiopharmaceuticals in Nuclear Medicine: Recent Developments for SPECT and PET Studies

**DOI:** 10.1155/2014/426892

**Published:** 2014-12-21

**Authors:** Bianca Gutfilen, Gianluca Valentini

**Affiliations:** ^1^Department of Radiology, Universidade Federal do Rio de Janeiro, Hospital Universitário Clementino Fraga Filho, Rua Professor Rodolpho Rocco, 255 Cidade Universitária, Ilha do Fundão, 21941-913 Rio de Janeiro, RJ, Brazil; ^2^Advanced Center Oncology Macerata (ACOM), Località Cavallino, 62010 Montecosaro, Italy

Nuclear medicine is returning to its origin by studying more and more metabolic signals using new positron or single-photon-emitting radiopharmaceuticals. The history of nuclear medicine over the past 50 years highlights the strong link between investments in chemistry and the development of radionuclides and radiolabeled compounds. In fact, one can trace the major advances in nuclear medicine directly to research in chemistry. These advances have had a major impact on the practice of health care. According to the Society of Nuclear Medicine, 20 million nuclear medicine procedures using radiopharmaceuticals and imaging instruments are carried out in hospitals in the United States alone each year to diagnose disease and to deliver targeted treatments. These techniques have also been adopted by basic and clinical scientists in different fields (infection, immunology, gastroenterology, cardiology, oncology, neurology, psychiatry, and others) for diagnosis as well as for scientific tools.

Many groups of research are now developing radiopharmaceuticals as biomarkers for new drug targets to facilitate the entry of their new drugs into the practice of health care and to objectively examine drug efficacy at a particular target relative to clinical outcome. This has created a demand for new radiopharmaceuticals and a corresponding need for scientists who are trained to develop them.

The traditional lack of techniques suitable for* in vivo* imaging has induced a great interest in molecular imaging for preclinical research. Nevertheless, its use spreads slowly due to the difficulties to justify the high cost of the current dedicated preclinical scanners. An alternative for lowering the costs is to repurpose old clinical gamma-cameras to be used for preclinical imaging. In this paper P. Aguiar et al. have assessed the performance of a portable device that is working coupled to a single-head clinical gamma-camera and have presented their preliminary experience in several small animal applications. Their findings, based on phantom experiments and animal studies, provided an image quality, in terms of contrast-noise trade-off, comparable to dedicated preclinical pinhole-based scanners. They suggest that their device can offer an opportunity for recycling the widespread availability of clinical gamma-cameras innuclear medicine departments to be used in small animal SPECT imaging contributing to spreading of the use of preclinical imaging within institutions on tight budgets.

Molecular imaging using single-photon (gamma) imaging (SPECT) and positron emission tomography (PET) based approaches is promising tools for noninvasive diagnosis of acute allograft rejection (AR). Given the importance of renal transplantation and the limitation of available donors, detailed analysis of factors that affect transplant survival is important. Episodes of acute allograft rejection are a negative prognostic factor for long-term graft survival. Invasive core needle biopsies are still the gold standard in rejection diagnostics. Nevertheless, they are cumbersome to the patient and carry the risk of significant graft injury. Notably, they cannot be performed on patients taking anticoagulant drugs. Therefore, a noninvasive tool assessing the whole organ for specific and fast detection of acute allograft rejection is desirable. H. Pawelski et al. have reviewed SPECT- and PET-based approaches for noninvasive molecular imaging-based diagnostics of acute transplant rejection.

Nuclear cardiology has experienced exponential growth within the past four decades with converging capacity to diagnose and influence management of a variety of cardiovascular diseases. SPECT myocardial perfusion imaging (MPI) with technetium-99m radiotracers or thallium-201 has dominated the field; however new hardware and software designs that optimize image quality with reduced radiation exposure are fuelling a resurgence of interest at the preclinical and clinical levels to expand beyond MPI. Other imaging modalities including PET and MRI continue to emerge as powerful players with an expanded capacity to diagnose a variety of cardiac conditions. At the forefront of this resurgence is the development of novel target vectors based on an enhanced understanding of the underlying pathophysiological process in the subcellular domain. Molecular imaging with novel radiopharmaceuticals engineered to target a specific subcellular process has the capacity to improve diagnostic accuracy and deliver enhanced prognostic information to alter management. O. O. Sogbein et al. have reviewed the recent advancements in radiotracer development for SPECT and PET MPI, autonomic dysfunction, apoptosis, atherosclerotic plaques, metabolism, and viability. The relevant radiochemistry, preclinical and clinical development, and molecular imaging with emerging modalities such as cardiac MRI and PET-MR have also been discussed.

Until recently, iodine-124 was not considered to be an attractive isotope for medical applications owing to its complex radioactive decay scheme, which includes several high-energy gamma rays. However, its unique chemical properties and convenient half-life of 4.2 days indicated it would be only a matter of time for its frequent application to become a reality. The development of new medical imaging techniques, especially improvements in the technology of PET such as the development of new detectors and signal processing electronics, has opened up new prospects for its application. With the increasing use of PET in medical oncology, pharmacokinetics, and drug metabolism, ^124^I-labeled radiopharmaceuticals are now becoming one of the most useful tools for PET imaging, and owing to the convenient half-life of I-124 they can be used in PET scanners far away from the radionuclide production site.

^124^Iodine (^124^I) is particularly attractive for* in vivo *detection and quantification of longer-term biological and physiological processes; the long half-life of ^124^I is especially suited for prolonged time* in vivo *studies of high molecular weight compounds uptake. Numerous small molecules and larger compounds like proteins and antibodies have been successfully labeled with ^124^I. Advances in radionuclide production allow the effective availability of sufficient quantities of ^124^I on small biomedical cyclotrons for molecular imaging purposes. Radioiodination chemistry with ^124^I relies on well-established radioiodine labeling methods, which consists mainly in nucleophilic and electrophilic substitution reactions. G. L. Cascini et al. have discussed all iodine radioisotopes application focusing on ^124^I that seems to be the most promising for its half-life, radiation emissions, and stability, allowing several applications in oncological and nononcological fields.

^64^Cu-Labeled molecules are promising imaging agents for PET due to the favorable nuclear characteristics of the isotope (*t*1/2 = 12.7 h, *β*+ 17.4%, *E*_max⁡_ = 0.656 MeV,  *β*− 39%, *E*_max⁡_ = 0.573 MeV) and its availability in high specific activity. The longer physical half-life of ^64^Cu compared to ^11^C (*t*1/2 = 20 min) and 18F (*t*1/2 = 110 min) enables imaging at delayed time points, which allows sufficient time for clearance from background tissues, resulting in increased image contrast, particularly for targeting agents that demonstrate long circulation times such as antibodies and nanoparticles. Moreover, ^64^Cu-based PET radiotracers have demonstrated efficacy for radioimmunotherapy comparable to that for the strictly therapeutic radionuclide, ^67^Cu (*t*1/2 = 61.5 h, *β*− 100%, *E*_max⁡_ = 0.121 MeV). Accordingly, ^64^Cu could be used for imaging and therapy concurrently.

Copper (Cu) is an important trace element in humans; it plays role as a cofactor for numerous enzymes and other proteins crucial for respiration, iron transport, metabolism, cell growth, and hemostasis. Natural copper comprises two stable isotopes, ^63^Cu and ^65^Cu, and 5 principal radioisotopes for molecular imaging applications (^60^Cu, ^61^Cu, ^62^Cu, and ^64^Cu) and* in vivo* targeted radiation therapy (^64^Cu and ^67^Cu). The two potential ways to produce Cu radioisotopes concern the use of the cyclotron or the reactor. A noncopper target is used to produce non-carrier-added Cu thanks to a chemical separation from the target material using ion exchange chromatography achieving a high amount of radioactivity with the lowest possible amount of nonradioactive isotopes. In recent years Cu isotopes have been linked to antibodies, proteins, peptides, and nanoparticles for preclinical and clinical research; pathological conditions that influence Cu metabolism such as Menkes syndrome, Wilson disease, inflammation, tumor growth, metastasis, angiogenesis, and drug resistance have been studied. A. N. Asabella et al. have discussed all Cu radioisotopes application focusing on ^64^Cu and in particular its form ^64^CuCl_2_ that seems to be the most promising for its half-life, radiation emissions, and stability with chelators, allowing several applications in oncological and nononcological fields.

Although neurological ailments continue to be some of the main causes of disease burden in the world, current therapies such as pharmacological agents have limited potential in the restoration of neural functions. Cell therapies, firstly applied to treat different hematological diseases, are now being investigated in preclinical and clinical studies for neurological illnesses. However, the potential applications and mechanisms for such treatments are still poorly comprehended and are the focus of permanent research. In this setting, noninvasive* in vivo *imaging allows better understanding of several aspects of stem cell therapies. Amongst the various methods available, radioisotope cell labeling has become one of the most promising since it permits tracking of cells after injection by different routes to investigate their biodistribution. A significant increase in the number of studies utilizing this method has occurred in the last years. P. H. Rosado-de-Castro et al. have reviewed the different radiopharmaceuticals, imaging techniques, and findings of the preclinical and clinical reports published up to now. Moreover, they have discussed the limitations and future applications of radioisotope cell labeling in the field of cell transplantation for neurological diseases.

W. Robeson et al. have demonstrated that, for dopaminergic radiotracers, 18F-FDOPA and 18F-FPCIT, the urinary bladder is the critical organ. As these tracers accumulate in the basal ganglia (BG) with high affinity and long residence times, radiation dose to the BG may become significant, especially in normal control subjects. They have performed dynamic PET measurements using 18F-FPCIT in normal adult subjects to determine if in fact the BG, although not a whole organ but a well-defined substructure, receives the highest dose. They have concluded that, for some normal subjects studied with F-18 or long half-life radionuclide, the BG may exceed bladder dose and become the critical structure.

These papers represent important observations into different topics related to recent developments for SPECT and PET studies. We hope that this special issue reaches researches all over the world who deal with this field.

